# In vivo binding of a tau imaging probe, [^11^C]PBB3, in patients with progressive supranuclear palsy

**DOI:** 10.1002/mds.27643

**Published:** 2019-03-20

**Authors:** Hironobu Endo, Hitoshi Shimada, Naruhiko Sahara, Maiko Ono, Shunsuke Koga, Soichiro Kitamura, Fumitoshi Niwa, Shigeki Hirano, Yasuyuki Kimura, Masanori Ichise, Hitoshi Shinotoh, Ming Rong Zhang, Satoshi Kuwabara, Dennis W. Dickson, Tatsushi Toda, Tetsuya Suhara, Makoto Higuchi

**Affiliations:** ^1^ Department of Functional Brain Imaging Research (DOFI), Clinical Research Cluster, National Institute of Radiological Sciences (NIRS) National Institutes for Quantum and Radiological Science and Technology (QST) Chiba Chiba Japan; ^2^ Division of Neurology Kobe University Graduate School of Medicine Kobe Hyogo Japan; ^3^ Department of Neuroscience Mayo Clinic Jacksonville Florida USA; ^4^ Department of Psychiatry Nara Medical University Kashihara Japan; ^5^ Department of Neurology Kyoto Prefectural University of Medicine Kyoto Japan; ^6^ Department of Neurology, Graduate School of Medicine Chiba University Chiba Japan; ^7^ Department of Clinical and Experimental Neuroimaging, Center for Development of Advanced Medicine for Dementia National Center for Geriatrics and Gerontology Obu Japan; ^8^ Neurology Chiba Clinic Chiba Japan; ^9^ Department of Radiopharmaceuticals Development Clinical Research Cluster, NIRS, QST Chiba Japan; ^10^ Department of Neurology University of Tokyo Graduate School of Medicine Tokyo Japan

**Keywords:** imaging, movement disorders, progressive supranuclear palsy, tau, tau imaging

## Abstract

**Background:**

[^11^C]pyridinyl‐butadienyl‐benzothiazole 3 is a PET imaging agent designed for capturing pathological tau aggregates in diverse neurodegenerative disorders, and would be of clinical utility for neuropathological investigations of PSP.

**Objectives:**

To explore the usefulness of [^11^C]pyridinyl‐butadienyl‐benzothiazole 3/PET in assessing characteristic distributions of tau pathologies and their association with clinical symptoms in the brains of living PSP patients.

**Methods:**

We assessed 13 PSP patients and 13 age‐matched healthy control subjects. Individuals negative for amyloid β PET with [^11^C]Pittsburgh compound B underwent clinical scoring, MR scans, and [^11^C]pyridinyl‐butadienyl‐benzothiazole 3/PET.

**Results:**

There were significant differences in binding potential for [^11^C]pyridinyl‐butadienyl‐benzothiazole 3 between PSP patients and healthy control subjects (*P* = 0.02). PSP patients exhibited greater radioligand retention than healthy control subjects in multiple brain regions, including frontoparietal white matter, parietal gray matter, globus pallidus, STN, red nucleus, and cerebellar dentate nucleus. [^11^C]pyridinyl‐butadienyl‐benzothiazole 3 deposition in frontoparietal white matter, but not gray matter, was correlated with general severity of parkinsonian and PSP symptoms, whereas both gray matter and white matter [^11^C]pyridinyl‐butadienyl‐benzothiazole 3 accumulations in the frontoparietal cortices were associated with nonverbal cognitive impairments. Autoradiographic and fluorescence labeling with pyridinyl‐butadienyl‐benzothiazole 3 was observed in gray matter and white matter of PSP motor cortex tissues.

**Conclusions:**

Our findings support the in vivo detectability of tau fibrils characteristic of PSP by [^11^C]pyridinyl‐butadienyl‐benzothiazole 3/PET, and imply distinct and synergistic contributions of gray matter and white matte tau pathologies to clinical symptoms. [^11^C]pyridinyl‐butadienyl‐benzothiazole 3/PET potentially provides a neuroimaging‐based index for the evolution of PSP tau pathologies promoting the deterioration of motor and cognitive functions. © 2019 The Authors. *Movement Disorders* published by Wiley Periodicals, Inc. on behalf of International Parkinson and Movement Disorder Society.

PSP is a neurodegenerative disorder clinically characterized by vertical gaze palsy, early postural instability with falls, axial rigidity, pseudobulbar palsy, and mild cognitive impairment.^1^, ^2^, ^3^ PSP is pathologically known as tauopathy and is characterized by deposits of tau isoforms with four repeat domains (4RTs) in neurons and glia cells including axonal tracts, in clear contrast with neuronal somatodentritic tau inclusions composed of all six isoforms along with amyloid β (Aβ) plaques in Alzheimer's disease (AD).^4^ As with the other sporadic and hereditary tauopathies, tau accumulation has been implicated in neurodegeneration and clinical symptoms in PSP.^5^, ^6^ Indeed, abundant tau lesions accompanied by neuronal loss and gliosis are found in neocortical and subcortical gray matter (GM) portions (e.g., precentral gyrus, striatum, globus pallidus, subthalamic nucleus, red nucleus, SN, and cerebellar dentate nucleus), and these structures are associated with clinical features characteristic of PSP.^2^, ^3^, ^7^ These findings have suggested involvements of distinct segments of the brain, including neocortical GM and subcortical nuclei, in neurodegenerative tau pathologies, whereas tau‐bearing anatomical structures associated with symptomatic manifestations and progressions are yet to be identified.

We have developed [^11^C]pyridinyl‐butadienyl‐benzothiazole 3 ([^11^C]PBB3), a PET radioligand with high affinity and specificity for tau assemblies. Both in vitro and in vivo assessments have indicated that [^11^C]PBB3 is capable of detecting diverse tau conformers, such as tau fibrils in AD and corticobasal degeneration.^6^ Other tau radioligands have also been applied to the detection of tau lesions in patients with PSP,^8^, ^9^, ^10^, ^11^, ^12^, ^13^, ^14^, ^15^ but evidence for the ability of PET imaging with these radiocompounds to separate PSP subjects from controls as well as the relationships between PET signals and clinical severity of PSP are still inconclusive. In addition, recent works have documented that in vivo [^18^F]THK5351 retention is affected by its off‐target binding to monoamine oxidase‐B (MAO‐B) expressed in astrocytes.^16^ Although in vivo [^18^F]AV‐1451 retention was shown to correlate with severity of PSP in a previous report,^11^ no such relationship was demonstrated in another study with a larger sample size.^17^ Moreover, a neuropathological assay of 2 PSP cases who had undergone a tau PET scan with [^18^F]AV‐1451 indicated no overt correlations between in vivo PET signals and the regional abundance of fibrillary tau aggregates.^13^ Our recent autoradiographic, fluorescence microscopic and tissue homogenate binding assays have demonstrated tight in vitro binding of [^11^C]PBB3 to wide‐ranging tau pathologies, including PSP tau lesions, relative to [^18^F]AV‐1451,^18^ whereas the in vivo performance of [^11^C]PBB3 in clinical PET assays of PSP patients is yet to be validated. It should be noted that insoluble tau filaments in PSP brains could be less abundant than those in AD brains, and the possible presence of tau inclusions in the cerebellum in PSP may lead to an underestimation of tau pathologies when analyzing PET data using the cerebellum as a reference tissue devoid of tau fibrils. To address this issue, we have recently applied a new method to generate a reference cluster by pooling voxels with low radioligand retention.^19^

The goal of the present study was to elucidate the distribution of [^11^C]PBB3‐PET–detectable tau lesions in PSP patients, followed by the localization of tau tracer accumulations correlated with the severity of clinical symptoms. Identities of PBB3 signals were further analyzed by autoradiographic and fluorescence labeling of brain sections with this compound in conjunction with histochemical and immunohistochemical assays.

## Patients and Methods

### Subjects

Thirteen PSP patients were recruited from Chiba University Hospital and affiliated hospitals between July 2012 and October 2015. Diagnosis of PSP was based on the International Parkinson and Movement Disorder Society clinical diagnostic criteria for PSP.^3^ The full study protocol, including detailed inclusion and exclusion criteria, are available on our institutional website (http://www.nirs.qst.go.jp/seika/brain/pdf/PBB3_Clinical_Study_Plan_jpn.pdf). Fourteen age‐matched healthy control subjects (HCs) without medical histories of abnormalities by clinical examinations were recruited from the volunteer association of the National Institute of Radiological Sciences (NIRS). We also incorporated a subset of [^11^C]PBB3‐PET data obtained from young HCs in a separate clinical study,^20^ primarily for the purpose of constructing a histogram of radioligand binding on a voxel‐by‐voxel basis (see eMethods in the Supporting Information for details).

The subjects underwent neurological examinations, including UPDRS‐III and PSP Rating Scale (PSPRS), for assessing motor symptoms and psychological evaluation, including Mini‐Mental State Examination (MMSE), Clinical Dementia Rating Scale (CDR), and Wechsler Memory Scale Revised Logical Memory II (WMSR LM‐II), for assessing cognitive and functional impairment, Frontal Assessment Battery (FAB) for assessing frontal dysfunction, and Raven's Coloured Progressive Matrices (RCPM) for assessing nonverbal cognitive impairment. RCPM reflects receptive vocabulary and visuospatial measures, and it can reportedly be conducted for subjects with severe motor impairments.^21^, ^22^ Clinical scores of PSPRS, FAB, CDR Sum of Boxes (CDR‐SOB), WMSR‐LM II, and RCPM were available in 10 (subcategory = 9), 12, 12, 9, and 11 PSP patients, respectively, and all scores except PSPRS were obtained from each of the HCs. Subjects aged >60 years with CDR‐SOB = 0 and WMSR LM‐II percentile >40 were recruited as HCs.

The Ethics and Radiation Safety Committees of the NIRS, Chiba, Japan approved this study. The study was registered with the UMIN Clinical Trials Registry (UMIN‐CTR; number 000009863).

### Demographic and Clinical Features of Participants

One of 14 age‐matched HCs (7%) and none of 13 PSPs were judged as Aβ positive by visual assessment of PET images acquired with [^11^C]Pittsburgh Compound‐B ([^11^C]PiB), which were generated by voxel‐based calculation of standardized uptake value ratio (SUVR) between the cerebral cortex and cerebellum at 50 to 70 minutes after an intravenous injection of [^11^C]PiB. The Aβ‐positive HC was excluded from further analyses. Thirteen PSP patients (age, 71 ± 8 years; men/women: 9/4) and 13 age‐matched HCs (age, 68 ± 4 years; men/women: 9/4) were incorporated in the group comparison. The PSP group consisted of 13 probable PSP with a mean disease duration of 5 ± 3 years, and all these patients presented a clinical phenotype of classical PSP/Richardson's syndrome.

Demographic and pertinent clinical characteristics of the subjects, including neurological and neuropsychological test scores, are shown in Table 1. There was no significant difference in age and sex between the HC and PSP groups (*P* = 0.30 and *P* > 0.99, respectively). Significant differences were observed in UPDRS motor subscale total, MMSE, FAB, CDR‐SOB, WMSR LM‐II, and RCPM scores between the two groups (*P* < 0.001 for each score).

**Table 1:** Demographic and pertinent clinical characteristics of subjects

Clinical Characters and Scores	HCs	PSPs	*P* Value
No. of subjects	13	13	
Age, mean (SD), y	68.0 (4.3)	71.3 (7.7)	0.30
Sex (male/female)	9/4	9/4	>0.99
Disease duration, mean (SD), y		4.9 (2.9)	
UPDRS (0–108), mean (SD)	0.9 (1.3)	43.9 (19.5)	<0.001
PSPRS (0–100), mean (SD)	N.E.	47.4 (19.2) (n = 10)	
MMSE (0–30), mean (SD)	29.0 (1.2)	16.7 (6.4)	<0.001
FAB (0–18), mean (SD)	17.3 (0.8)	9.0 (4.5) (n = 12)	<0.001
CDR‐SOB (0–18), mean (SD)	0	6.9 (4.7) (n = 12)	<0.001
WMSR LM‐II, mean (SD), percentile	71.8 (18.9)	5.9 (5.9) (n = 9)	<0.001
RCPM (0–36), mean (SD)	31.4 (3.6)	18.6 (8.7) (n = 11)	<0.001

N.E.: not examined.

### MRI Studies

Subjects underwent an MRI scan either on a 3‐Telsa (T) Signa HDxt (GE Healthcare, Little Chalfont, UK), or a 3 T MAGNETOM Verio (SIEMENS, Erlangen, Germany). T_1_‐weighted images were taken for coregistration and segmentation of PET images (Signa HDxt: axial orientation as 1‐mm‐thick sections, echo time [TE] 2.848 ms, repetition time [TR] 6.996 ms, flip angle 8.0 degrees, inversion time [TI] 900 ms, field of view [FOV] 260 mm, matrix size 256 × 256 × 166; MAGNETOM Verio: sagittal orientation as 1‐mm‐thick sections, TE 1.95 ms, TR 2300 ms, flip angle 9.0 degrees, TI 900 ms, FOV 250 mm, matrix size 512 × 512 × 176). MR images were segmented into GM, white matter (WM), and nonbrain space (cerebrospinal fluid, skull, and meninges) using SPM12 (Wellcome Department of Imaging Neuroscience, London, United Kingdom).

### PET Studies

Radiosynthesis of [^11^C]PBB3 and [^11^C]PiB was carried out as described elsewhere (see eMethods in the Supporting Information for details).^6^, ^19^, ^23^ PET scans were performed with an ECAT EXACT HR+ scanner (Siemens/CTI PET Systems). Detailed PET scanning protocols were described previously.^6^, ^19^, ^23^ Each subject serially received two 70‐minute PET scans (frames, 3 × 20 and 3 × 40 sec and 1 × 1, 2 × 3, 5 × 6, and 3 × 10 minutes) after intravenous injection of [^11^C]PBB3 (440 ± 82 MBq) followed by [^11^C]PiB (416 ± 64 MBq) 2.0 to 2.5 hours apart on the same day.

### PET Quantification of Tau Pathology

To quantify the regional distribution of tau pathologies, we generated parametric maps of a binding potential (BP*_ND_: a binding parameter that reflects the density of tau deposits) for [^11^C]PBB3 as described previously.^23^ For this quantification, we used an optimized GM reference instead of a conventional cerebellar cortical reference to minimize inclusion of tau‐positive areas in the reference tissue, according to a previously described method with some modifications.^19^ Briefly, the covering range of BP*_ND_ for picking up reference voxels was determined in young HCs, and reference voxels of GM were extracted from the image data of each subject using the range defined above. Finally, parametric BP*_ND_ images were generated by a reference tissue model with these individually pooled reference voxels.^23^ Extractions of reference voxels are exemplified in the Supporting Information (Supporting Information Figure S1), and descriptions of the current procedure to define reference tissue voxels are also provided in the Supplement (eMethods). Volumes of interest (VOIs) are defined on parametric images manually or semiautomatically (see eMethods and Supporting Information Figure S2 for details).

Image data analysis was performed with PMOD3.6 (PMOD Technologies Ltd, Zürich, Switzerland) and SPM12 software (Wellcome Department of Imaging Neuroscience).

### Autoradiographic, Histochemical, and Immunohistochemical Labeling of Brain Sections

Postmortem brain sections from 2 patients pathologically diagnosed as PSP (86‐year‐old male and 71‐year‐old male) were obtained from the Mayo Clinic Brain Bank. Both cases were Braak neurofibrillary tangle stage III and Thal amyloid phase 0, indicating that notable AD‐spectrum changes were absent in these cases. Brain autopsies were performed upon consent of the legal next of kin or individual with power of attorney. Studies of autopsy samples were considered exempt from human subject research by the Mayo Clinic Institutional Review Board. Tissues were fixed in 10% neutral buffered formalin and embedded in paraffin blocks.

In vitro autoradiography was conducted on PSP brain sections, and the methodological details of this assay are described in the Supporting Information (eMethods). We also performed triple staining of adjacent brain slices with PBB3 fluorescence, AT8 immunohistochemistry, and Gallyas silver (GS) impregnation (see eMethods in the Supporting Information for details). Briefly, in vitro fluorescence staining was performed with PBB3, followed by reaction with a monoclonal antibody against phosphorylated tau and AT8 (1:250; Thermo Scientific, Rockford, IL). Finally, GS staining was applied to the same sections.

### Statistical Analysis

We used multivariate analysis of variance for comparison of PET data between HCs and PSP patients. For group analysis of demographic data between these groups, we adopted Fisher's exact test (sex) and Mann‐Whitney U test with Bonferroni correction (rest of the data). For examining correlations between BP*_ND_ and clinical scores in PSP patients, we used Spearman's rho test. For data comparison, we obtained false discovery rate *q* values calculated using the Benjamini‐Hochberg method to accommodate multiple comparisons. The range of statistical significance was defined as *P* ≤ 0.05. We used IBM SPSS Statistics (version 22; IBM, Armonk, NY) for all statistical analyses. All values are reported as mean ± standard deviation.

## Results

### [^11^C]PBB3 Binding in PSP Patients Versus HCs

**Figure 1:**
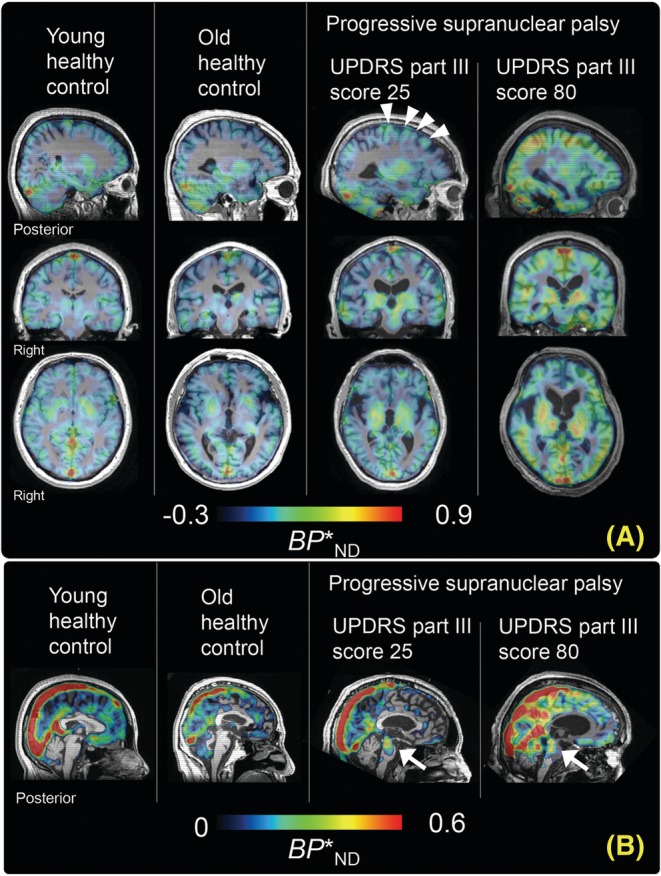
Representative parametric images of [^11^C]PBB3 BP*_ND_ in 1 young HC (age, 35 years) and 1 old (age, 75 years) HC and 2 patients with PSP with mild (age, 57 years; UPDRS, 25 points) and severe (age, 75 years; UPDRS, 80 points) symptoms (focusing on neocortex [A] and brainstem [B]). PET data are fused on corresponding T_1_‐weighted MR images. Arrows indicate increased radioligand binding in the midline area of the PSP midbrain. Arrow heads show increased radioligand binding in the lateral area of the PSP frontal lobe. Please note that there is a difference in dynamic range between (A) and (B) because values of [^11^C]PBB3 BP*_ND_ in white matter including brain stem are smaller than those in gray matter. Binding potential (BP*_ND_; binding parameter that directly reflects tau density).

Parametric BP*_ND_ images illustrated minimal age‐related alterations of [^11^C]PBB3 binding in HCs, despite higher radioligand retention in the caudate and thalamus than in other areas. Increased BP*_ND_ was found in several neocortical and subcortical regions, such as the pericentral cortices, globus pallidus, and brain stem nuclei, of a patient with relatively mild PSP, then spreading to extensive brain areas with disease progression (Fig. 1). Of note were the markedly intensified radioligand accumulations in the midbrain of PSP patients from a relatively early stage in clear distinction from the minimal radiosignal retentions in this area of HCs (Fig. 1B). There were differences in BP*_ND_ values between PSP patients and HCs (Pillai's trace, V = 1.00; *F*_(23,2)_ = 52.72; *P* = 0.02; Supporting Information Table S1).

**Figure 2:**
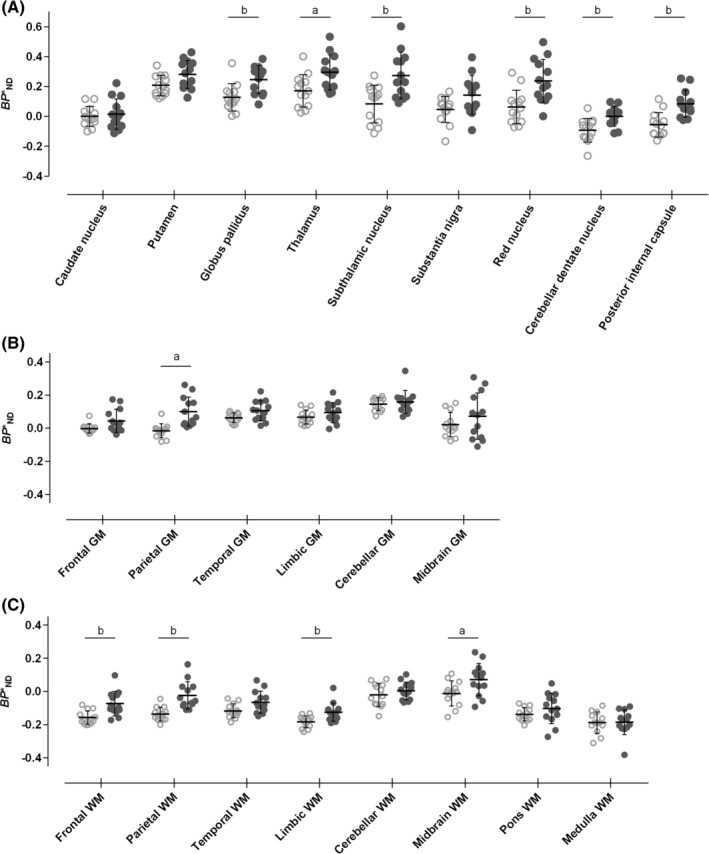
(A) Plots of BP*_ND_ in subcortical nuclear VOI of HCs (hollow circles) and PSP patients (solid circles). Definition of VOI is provided in Supporting Information Figure S2A. Error bars represent mean values ± SD. a, *P* ≤ 0.05; b, *P* ≤ .01. (B) Plots of BP*_ND_ in neocortical, cerebellar, and midbrain GM VOI of HCs (hollow circles) and PSP patients (solid circles). Definition of regions of interest is provided in Supporting Information Figure S2B. Error bars represent mean values ± SD. a, *P* ≤ 0.05. (C) Plots of BP*_ND_ in neocortical, cerebellar, and brainstem WM VOI of HCs (hollow circles) and PSP patients (solid circles). Definition of VOI is provided in Supporting Information Figure S2C. Error bars represent mean values ± SD. a, *P* ≤ 0.05; b, *P* ≤ 0.01. Binding potential (BP*_ND_; binding parameter that directly reflects tau density).

BP*_ND_ values in VOIs manually defined on subcortical nuclei were evident in a comparison of PSP patients and HCs, and six of nine subcortical regions, including the globus pallidus, STN, and cerebellar dentate nucleus, displayed a marked elevation of BP*_ND_, with Z scores ranging from 1.13 (thalamus) to 1.72 (posterior internal capsule) (Fig. 2A; Supporting Information Table S1).

PSP patients exhibited greater radioligand retention than HCs in VOIs semiautomatically defined on GMs, and BP*_ND_ values in parietal region in PSP cases were significantly higher than those in HCs, with 2.75 of Z score (Fig. 2B; Supporting Information Table S1). Trend of high BP*_ND_ values was also observed in frontal and temporal GMs of PSP patients with 0.18 and 0.17 of effect size, and 1.75 and 1.43 of Z scores, respectively (Supporting Information Table S1).

A significant increase of BP*_ND_ values in WM VOIs of PSP patients was also observed, and significant differences in these parameter values between the two groups were noted in the frontal, parietal, limbic, and midbrain WMs, with 2.10, 2.72, 1.65, and 1.12 of Z scores, respectively (Fig. 2C; Supporting Information Table S1). Meanwhile, alterations of [^11^C]PBB3 BP*_ND_ in PSP subjects were not detectable by PET in limbic GM and infratentorial GM and WM except midbrain WM. A receiver operating characteristic curve analysis of regional BP*_ND_ values for [^11^C]PBB3 indicated that measurements of BP*_ND_ in parietal GM allow discriminations between PSP cases and HCs with sensitivity and specificity exceeding 90% (Supporting Information Table S2; Supporting Information Fig. S3).

**Figure 3:**
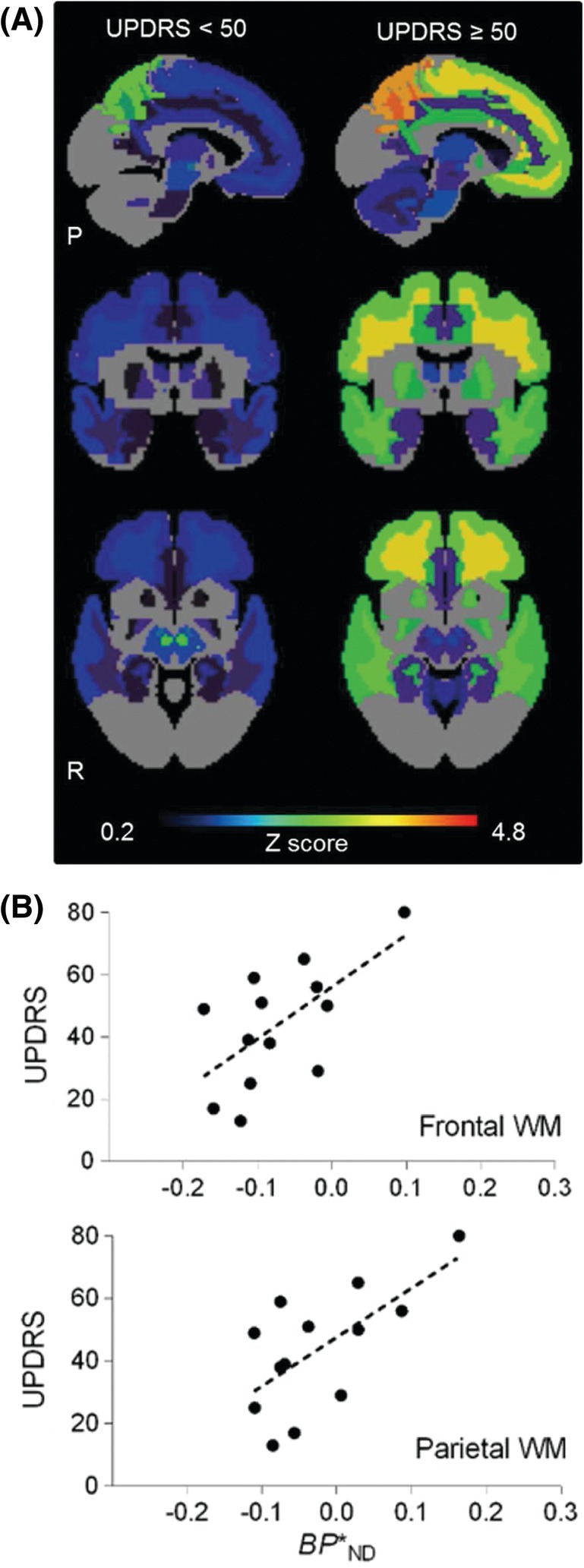
(A) Maps illustrate Z scores for BP*_ND_ values in PSP patients with earlier (UPDRS scores <50 points, n = 7, left column) and more advanced (UPDRS ≥50 points, n = 6, right column) relative to HCs using the rainbow color scale shown at the bottom. (B) Positive correlations between motor impairments (UPDRS scores) and BP*_ND_ in PSP patients. Scatter plots of BP*_ND_ in frontal (*r*_*s*_ = 0.6, *P* = 0.05) and parietal (*r*_*s*_ = 0.6, *P* = 0.03) WM VOIs against UPDRS scores in PSP patients. Dashed lines represent regressions. Binding potential (BP*_ND_; binding parameter that directly reflects tau density); healthy control subjects (HCs), P, posterior; R, right (R).

We then divided PSP patients into subjects with milder (UPDRS scores <50 points) and more advanced (UPDRS scores ≥50 points) motor impairments according to median score (49 points) to obtain an adequate statistical power in each subgroup, and Z score maps for differences in BP*_ND_ values from HCs in multiple VOIs were generated (Fig. 3A). Elevation of Z scores was noticeable at an earlier stage in neocortical GM/WM and subcortical nuclei and was expanded to the cerebellum with disease progression. In the neocortex, it is noteworthy that stage‐dependent increases of Z scores in WM were more remarkable than those in GM. By contrast, Z scores in the midbrain did not increase with the advancement of PSPs.

### Correlations Between [^11^C]PBB3 Binding and Clinical Symptoms

Among PSP patients, there were significant positive correlations between UPDRS scores and BP*_ND_ values in WM of frontal and parietal regions (*r*_*s*_ = 0.6, *P* = 0.05, *q* = 0.05 and *r*_*s*_ = 0.6, *P* = 0.03, *q* = 0.04, respectively; Supporting Information Fig. S3 Supporting Information Table S3), although no such correlations were observed in neocortical GM and subcortical nuclei involved in the extrapyramidal system (*P* > 0.05; data not shown). There were no correlations between total PSPRS scores and BP*_ND_ values in subcortical nuclei and neocortical GM/WM (*P* > 0.05), although scores of PSPRS subcategories were closely associated with BP*_ND_ values in GM and WM portions of several brain regions (Supporting Information Table S4).

BP*_ND_ values were correlated with nonverbal cognitions as scored by RCPM in GM/WM of frontal (*r*_*s*_ = –0.8, *P* = 0.004, *q* = 0.02/*r*_*s*_ = –0.7, *P* = 0.02, *q* = 0.04, respectively) and parietal (*r*_*s*_ = –0.7, *P* = 0.02, *q* = 0.03/*r*_*s*_ = –0.7, *P* = 0.01, *q* = 0.04, respectively) regions (Supporting Information Fig. S4 Supporting Information Table S3). Radioligand retention was not associated with RCPM scores in other areas (*P* > 0.05; data not shown).

### Autoradiographic and Histopathological Assays

**Figure 4:**
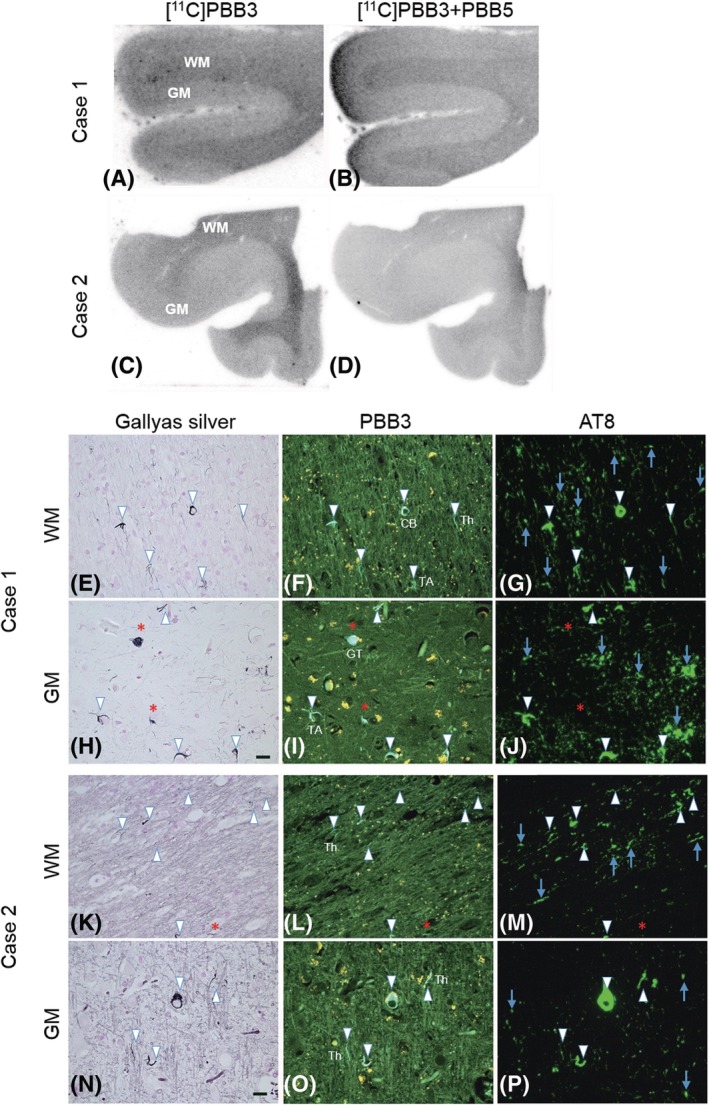
(A–D) Autoradiographic labeling of PSP motor cortex slices of case 1 (71‐year‐old male) with [^11^C]PBB3 in the absence (left) and presence (right) of excess nonradioactive PBB5. (E–P) Histochemical and immunohistochemical staining of PSP motor cortex slices of case 1 (71‐year‐old male) and case 2 (86‐year‐old male). Sections derived from case 1 are adjacent to those used for autoradiography. Panels display GS (left), PBB3 fluorescence (center), and AT8 immunofluorescence (light) staining of the same areas of WM (upper panels) and GM (lower panels). PBB3‐positive, silver‐positive inclusions are either AT8 positive (arrowheads) or AT8 negative (asterisks). These lesions are coexistent with putative immature tau aggregates that are AT8‐positive, but unlabeled with PBB3 and silver impregnation (arrows). Scale bar, 20 μm. CB, coiled body; GT, globose tangle; Th, thread; TA, tufted astrocyte.

Specific binding of [^11^C]PBB3 was additionally investigated by autoradiography of PSP brain sections. In line with in vivo PET results, autoradiographic labeling of WM with [^11^C]PBB3 was more intense than that of GM in motor cortex slices and was blocked by the addition of excess nonradioactive PBB5 (Fig. 4A–D).

Triple staining of adjacent tissue slices demonstrated colocalization of PBB3 fluorescence staining with GS‐ and AT8‐positive tau inclusions, consisting of globose tangles in neurons in GM, neuritic tau deposits and oligodendrocytic coiled bodies in WM, and tufted astrocytes in GM and WM (Fig. 4E–P). A small subset of neuronal tau inclusions was positive in PBB3 and GS staining, but was not AT8 positive, implying that these lesions were extracellular tau deposits dubbed “ghost tangles” lacking AT8 epitopes. These PBB3‐positive tau aggregates coexisted with PBB3‐negative, but AT8‐positive, tau lesions, which were presumed to be relatively immature tau fibrils.

## Discussion

[^11^C]PBB3‐PET has shown association of radioligand binding in the frontoparietal WM with clinical dysfunctions, possibly assisting staging of the disease in PSP. This is in profound contrast to the fibrillar tau lesions in AD, rather than those confined to GM, spreading from the hippocampal formation to extensive neocortical regions.^6^ Hence, the composition of tau isoforms and interactions of tau with Aβ and other nontau amyloidogenic protein species may determine localization of tau deposition and focal symptoms.

Distribution of tau pathologies in both cortical and subcortical structures of PSP brains in clear distinction from the topology of tau accumulations in AD raises a concern on the definition of reference tissue for quantification of radioligand binding. Indeed, it is known that the cerebellar cortex and dentate nucleus, midbrain, pons, thalamus, and basal ganglia are affected by tau depositions,^24^, ^25^, ^26^ impeding selection of a distinctive brain region devoid of tau ligand‐binding components as a reference. Our method to extract reference voxels based on the likelihood of the presence of binding elements has allowed an analysis of the radioligand kinetics even in such a condition, and advantages of this and similar reference tissue methodologies over a conventional technique using cerebellar GM references for quantification of tau PET data have recently been documented.^27^

Our results also highlight distinct significances of [^11^C]PBB3 binding in neocortical WM and subcortical nuclei. Increases of [^11^C]PBB3 binding in the majority of subcortical nuclei were detectable in PSP patients, and Z score for the difference in BP*_ND_ between patients with UPDRS scores <50 and HCs was highest in the brainstem among these structures (Figs. 1B and 3A). These findings implicate changes of nigral BP*_ND_ in PSP at a relatively early stage, and are consistent with the fact that postural instability with falls emerge at an early stage of PSP. Meanwhile, significant correlation of [^11^C]PBB3 binding with the severity of motor impairments was found almost exclusively in the frontal and parietal WM, indicating the applicability of BP*_ND_ in these areas to quantitative assessments of disease progression.

The reason for the lack of such correlations in other regions, including the SN, is yet to be clarified. Similarly, the area under the curve in ROC analyses for separation between PSP and HC groups was the highest in parietal VOIs, and either sensitivity or specificity for the group differentiation was below 80% in all other regions (Supporting Information Table S2). This finding is in contrast to a recent [^18^F]AV‐1451/PET study showing the best intergroup differentiation achieved using radioligand retentions in the globus pallidus.^17^ It is likely that damaged neurons along with PBB3‐positive tau inclusions are eliminated as the disease advances and marked atrophy attributed to tau‐induced neurotoxicity results in a partial volume effect on radioligand binding measures,^24^, ^28^, ^29^, ^30^ leading to underestimation of BP*_ND_ values for [^11^C]PBB3 in advanced PSP cases. Because [^11^C]PBB3 is promptly converted to metabolites after intravenous injection, its uptake in the brain is greatly dependent on the first‐pass extraction. In the frontal and temporal cortices and subcortical nuclei, which are profoundly involved in tau‐induced neurodegenerative processes, declines of regional cerebral blood flow could lead to decreases of the first‐pass extraction, giving rise to insufficient radioligand uptake and potential underestimation of radiosignal retentions in these areas. By contrast, such degenerative changes and consequent decline of the radioligand delivery were less prominent in the parietal cortex. Moreover, according to a previous report on pathological staging of PSP,^25^ it should also be considered that tau pathologies in the midbrain and pons become almost full blown at a moderate stage. It is yet to be examined by postmortem assays of subjects undergoing PET scans how precisely parietal [^11^C]PBB3 retentions reflect local tau accumulations, and collections of such data are ongoing.

The relationships between PET and clinical data obtained here may also implicate PET‐detectable tau pathologies in frontoparietal GM and WM in the evolution of different symptomatic domains. In light of previous postmortem studies, the accumulation of insoluble tau fibrils in WM is a histopathological and biochemical feature of PSP,^26^, ^28^, ^31^ and our autoradiographic and histochemical assays support the detectability of numerous neuronal and glial tau inclusions in WM. Since BP*_ND_ for [^11^C]PBB3 in frontal and parietal WM was intimately correlated with the motor deficits scored by UPDRS, it can be presumed that tau‐induced axonal injuries contribute to progressive deteriorations of motor functions. In accord with this notion, recent works with diffusion tensors and functional MRI suggested that WM tract disruptions in the cerebello‐thalamo‐cortical system may lead to clinical symptoms of PSP patients.^32^, ^33^ Notably, Agosta and colleagues reported that UPDRS scores in PSP patients were significantly correlated with the severity of WM tract damages in the corpus callosum.^34^ In consideration of the fact that nerve fibers traceable from the corpus callosum spread through extensive frontoparietal WM regions, the present PET findings point to tau fibrillogenesis in frontoparietal WM as a pathological process underlying callosal abnormalities. Although scores of PSPRS were not correlated with BP*_ND_ in any regions, statistical power such as sample size could influence the result. [^11^C]PBB3 binding in frontoparietal WM could more tightly correlate with motor symptoms of UPDRS part III (motor section) than PSPRS, consisting of the following six categories: daily activities, behavior, bulbar, ocular motor, limb motor, and gait/midline. Association of the radioligand binding in frontal WM with motor deficits implies focal symptoms induced by on‐site tau neurotoxicity. However, it is still unclear whether tau pathology in parietal WM has direct effects on motor functions. It is likely that apraxia is provoked by deteriorations of the motor engram conveyed from the parietal lobe to motor areas by association fibers, being superimposed on motor dysfunctions.^35^ Unlike motor impairments, cognitive deficits examined by RCPM were tightly correlated with [^11^C]PBB3 binding in frontoparietal GM and WM. Given that nonverbal cognitive dysfunctions of RCPM are known to be related to prefrontal and parietal cortices,^36^, ^37^ aggravations of cognitive subdomains may occur as a consequence of combined GM and WM tau pathologies along the clinical course of PSP.

An additional issue of in vivo tau imaging with [^11^C]PBB3 is the off‐target binding of this radioligand in the basal ganglia and midbrain, because high radioactivity retention was observed in both young and old HCs (Fig. 1). Our previous autoradiographic assays demonstrated only minimal nonspecific binding of [^11^C]PBB3 in the basal ganglia sections.^18^ More recently, an in vitro binding assay using human brain homogenates has shown cross‐reactivity of [^11^C]PBB3 with neither monoamine oxidase‐A nor MAO‐B.^38^ Another concern could be binding of [^11^C]PBB3 to non‐tau protein fibrils, such as α‐synuclein aggregates.^39^ As a previous autoradiographic report documented, α‐synuclein deposits with very high abundance in MSA brains could be captured by [^11^C]PBB3, whereas none of the α‐synuclein pathologies in dementia with Lewy bodies (DLB) brains were detectable by this radioligand.^39^ In addition, our preliminary analysis with DLB brain homogenates has indicated that the dissociation constant of [^11^C]PBB3 for α‐synuclein deposits is approximately 10‐fold larger than the value for tau aggregates (Ono and colleagues, unpublished data), suggesting that concomitant α‐synuclein pathology may have little influence on the regional binding of [^11^C]PBB3 in tauopathy brains. Meanwhile, possible cross‐reactivity of [^11^C]PBB3 with aggregated TAR DNA‐binding protein of 43 kDa (TDP‐43) was also indicated in a recent PET study^40^ and is yet to be further examined by histochemical, autoradiographic, and homogenate binding assays with brain tissues burdened with TDP‐43 deposits. It will also be required to analyze the abundance of TDP‐43 inclusions in PSP brains to estimate the specificity of [^11^C]PBB3 for tau fibrils in PSP cases.

Whereas the current results support the ability of [^11^C]PBB3 to capture PSP tau pathologies in living patients, an individual assessment as a diagnostic adjunct would require a radioligand yielding a higher contrast. Relative to old HCs, the retention of [^11^C]PBB3 estimated as (BP*_ND_ + 1), which is theoretically close to SUVR, in areas enriched with tau deposits in PSP, including the cerebellar dentate nucleus, midbrain, STN, and globus pallidus was 1.09‐ to 1.18‐fold in PSP patients. According to data presented by Schonhaut and colleagues,^17^ SUVRs for [^18^F]AV‐1451 in PSP patients in these areas are 1.03‐ to 1.16‐fold higher than control values. Hence, it is likely that these two radioligands produce similar contrasts for PSP tau aggregates, notwithstanding further needs for considerations of methodological differences between the two works. Although a previous study demonstrated that *in‐vitro* binding of [^11^C]PBB3 to 4‐RT pathologies is greater than that of [^18^F]AV‐1451,^18^ in vivo binding of these compounds may not markedly differ, primarily because of rapid metabolism of [^11^C]PBB3 lowering its uptake in the brain. Based on the current findings validating the chemical class of PBB3 for capturing 4RT fibrils, ^18^F‐labeled derivatives of PBB3 with a higher contrast for target lesions has been lately developed, and nonclinical and clinical PET evaluations of these compounds are underway.^41^

Despite links between regional [^11^C]PBB3 binding and clinical symptoms, there remains controversy regarding neurotoxic tau species, because both soluble oligomers and thickened fibrils of tau could exert neuronal insults. The presence of PBB3‐positive extracellular tau aggregates (Fig. 4) could be neuropathological evidence for the loss of neurons and/or glia bearing highly mature tau fibrils, whereas phosphorylated tau deposits undetectable by PBB3 could trigger degenerative processes.^42^, ^43^, ^44^ The neurotoxicity triggered by these two distinct tau assemblies has also been illustrated by our combined in vitro and in vivo investigations of tau transgenic mouse models with [^11^C]PBB3.^36^ Because these mature and immature tau accumulations coexisted in the same brain area of PSP brains, [^11^C]PBB3‐PET images should be expected to represent the occurrence of tau‐provoked toxicities.

## Conclusions

The utility of [^11^C]PBB3‐PET for detection of tau aggregates characteristic of PSP has been supported by the present data, implying clinicopathological correlations in specific brain areas including frontoparietal WM. Our results with the current tau PET imaging technique have demonstrated the potential applicability to a surrogate index for clinical advancements resulting from progressive tau accumulations.

## Author Roles

(1) Research Project: A. Conception and Design; B. Acquisition of Data; C. Analysis and Interpretation of Data; (2) Manuscript: A. Writing of the First Draft, B. Review and Critique; (3) Other: A. Statistical Design; B. Processing and Analysis of Pathological Specimens; C. Methods for Data Analysis; D. Synthesizing the Radioligands.

H.E.: 1A, 1B, 1C, 2A, 3A

H.Shimada: 1A, 2B

N.S.: 2B, 3B

M.O.: 2B, 3B

S.Koga: 2B, 3B

S.Kitamura: 1B, 1C, 2B

F.N.: 1B, 1C, 2B

S.H.: 1A, 2B

Y.K.: 2B, 3A, 3C

M.I.: 2B, 3A, 3C

H.Shinotoh: 1A, 2B

M.R.Z.: 2B, 3D

S.Kuwabara: 1A, 2B

D.W.D.: 2B, 3B

T.T.: 1A, 2B

T.S.: 1A, 2B

M.H.: 1A, 2B

## Financial Disclosures

M.H., T.S., H.S., and M.R.Z. hold a patent related to tau imaging agents used in this study. This work was supported by grants from Grants‐in‐Aid for Brain Mapping by Integrated Neurotechnologies for Disease Studies (Brain/MINDS; 15653129) to T.S. and M.H. and Research and Development Grants for Dementia (16768966) to M.H. from the Japan Agency for Medical Research and Development, the Japan Advanced Molecular Imaging Program and Grants‐in‐Aid for Young Scientists (A) (26713031) to H.S. and Scientific Research on Innovative Areas (“Brain Environment” 23111009 to M.H. and “Brain Protein Aging” 26117001 to N.S.) from the Ministry of Education, Culture, Sports, Science and Technology, Japan, and Mochida Memorial Foundation for Medical Pharmaceutical Research to H.S.

## Supporting information

Table S1 Supporting information

Figure S1 Supporting information

Appendix S1 Supporting information
