# Supplementary material for: In vivo binding of a tau imaging probe, [11C]PBB3, in patients with progressive supranuclear palsy
Source: Mov Disord. 2019 Mar 20;34(5):744–54. doi: 10.1002/mds.27643 (PMC6593859; doi:10.1002/mds.27643)
Supplement: Supplementary file 1 — Table S1 Supporting information [file MDS-34-744-s001.pdf]

Table for review only

	Frontal GM	Parietal GM	Temporal GM	Limbic GM	Midbrain GM	Cerebellum GM	Frontal WM	Parietal WM	Temporal WM	Limbic WM	Midbrain WM	PonsWM	Medulla WM	Cerebellum WM	Cauadte nuclus	Putamen	Globus pallidus	Thalamus	Subthalamic nucleus	Substantia nigra	Red nucleus	Cerebellar dentate nucleus	Posterior internal capsule
PSP1	−0.013	0.006	0.063	0.065	0.230	0.069	−0.110	−0.110	−0.097	−0.134	0.211	−0.040	−0.091	0.051	0.014	0.237	0.331	0.369	0.368	0.206	0.386	0.020	0.246
PSP2	0.000	0.047	0.030	−0.005	−0.067	0.190	−0.094	−0.038	−0.111	−0.143	−0.027	−0.151	−0.170	−0.049	−0.092	0.356	0.302	0.212	0.090	−0.092	0.002	−0.106	0.051
PSP3	0.033	0.066	0.107	0.058	−0.056	0.111	−0.113	−0.069	−0.081	−0.190	0.035	−0.166	−0.200	−0.054	−0.035	0.192	0.195	0.200	0.126	0.068	0.146	0.013	0.035
PSP4	0.041	0.149	0.160	0.107	0.185	0.162	−0.037	0.029	−0.003	−0.105	0.110	−0.011	−0.205	0.075	0.147	0.350	0.333	0.444	0.228	0.060	0.113	0.050	0.254
PSP5	−0.013	0.029	0.062	0.066	0.098	0.170	−0.123	−0.085	−0.134	−0.108	0.084	−0.080	−0.103	0.038	−0.063	0.221	0.154	0.181	0.171	0.196	0.192	−0.033	−0.007
PSP6	−0.038	0.022	0.015	0.017	−0.020	0.165	−0.105	−0.075	−0.131	−0.167	0.043	−0.113	−0.107	−0.015	0.002	0.289	0.148	0.297	0.276	0.023	0.209	−0.034	0.085
PSP7	0.010	0.014	0.051	0.043	0.012	0.111	−0.084	−0.075	−0.090	−0.126	0.115	−0.104	−0.226	0.024	−0.035	0.182	0.141	0.309	0.370	0.217	0.298	0.025	−0.019
PSP8	−0.014	0.030	0.093	0.091	−0.110	0.144	−0.172	−0.110	−0.148	−0.182	−0.057	−0.273	−0.382	−0.062	−0.112	0.126	0.081	0.159	0.118	0.034	0.108	−0.045	−0.024
PSP9	0.086	0.235	0.110	0.138	−0.076	0.087	−0.020	0.087	−0.039	−0.122	−0.092	−0.233	−0.159	0.012	−0.070	0.314	0.279	0.152	0.133	0.080	0.136	0.096	0.050
PSP10	0.116	0.100	0.159	0.148	0.080	0.137	−0.006	0.029	0.035	−0.131	0.033	−0.017	−0.216	−0.010	0.223	0.393	0.301	0.278	0.230	0.159	0.244	0.093	0.097
PSP11	0.164	0.183	0.166	0.157	0.269	0.180	−0.019	0.006	−0.039	−0.069	0.142	−0.055	−0.149	−0.065	0.065	0.202	0.297	0.404	0.454	0.306	0.418	−0.113	0.112
PSP12	0.032	0.153	0.127	0.116	0.092	0.185	−0.159	−0.056	−0.069	−0.159	0.099	−0.139	−0.222	−0.002	0.138	0.429	0.254	0.304	0.394	0.203	0.350	0.027	0.053
PSP13	0.174	0.261	0.222	0.215	0.307	0.346	0.097	0.164	0.067	0.021	0.237	0.049	−0.172	0.103	0.021	0.369	0.386	0.534	0.603	0.395	0.497	0.007	0.164
**average**	**0.044**	**0.100**	**0.105**	**0.094**	**0.073**	**0.158**	**−0.073**	**−0.023**	**−0.065**	**−0.124**	**0.072**	**−0.103**	**−0.185**	**0.004**	**0.016**	**0.282**	**0.246**	**0.295**	**0.274**	**0.143**	**0.238**	**0.000**	**0.084**
**SD**	**0.070**	**0.088**	**0.061**	**0.061**	**0.140**	**0.068**	**0.073**	**0.082**	**0.066**	**0.055**	**0.097**	**0.091**	**0.075**	**0.054**	**0.102**	**0.095**	**0.093**	**0.117**	**0.155**	**0.130**	**0.144**	**0.065**	**0.091**
**Z score**	**1.745**	**2.754**	**1.435**	**0.618**	**0.704**	**0.341**	**2.104**	**2.725**	**1.237**	**1.649**	**1.116**	**0.867**	**0.041**	**0.349**	**0.220**	**1.082**	**1.312**	**1.133**	**1.514**	**1.098**	**1.569**	**1.164**	**1.716**
**minimum**	**−0.038**	**0.006**	**0.015**	**−0.005**	**−0.110**	**0.069**	**−0.172**	**−0.110**	**−0.148**	**−0.190**	**−0.092**	**−0.273**	**−0.382**	**−0.065**	**−0.112**	**0.126**	**0.081**	**0.152**	**0.090**	**−0.092**	**0.002**	**−0.113**	**−0.024**
**maximum**	**0.174**	**0.261**	**0.222**	**0.215**	**0.307**	**0.346**	**0.097**	**0.164**	**0.067**	**0.021**	**0.237**	**0.049**	**−0.091**	**0.103**	**0.223**	**0.429**	**0.386**	**0.534**	**0.603**	**0.395**	**0.497**	**0.096**	**0.254**
HC1	−0.015	−0.008	0.026	0.013	−0.078	0.156	−0.191	−0.156	−0.186	−0.229	−0.120	−0.203	−0.282	−0.077	−0.101	0.121	0.088	0.037	−0.113	−0.065	−0.072	−0.140	−0.117
HC2	−0.020	−0.076	0.047	0.056	0.026	0.184	−0.103	−0.092	−0.092	−0.135	0.009	−0.083	−0.204	0.075	−0.016	0.275	0.357	0.247	0.200	0.084	0.009	−0.029	0.116
HC3	0.004	−0.002	0.062	0.083	−0.011	0.130	−0.203	−0.145	−0.153	−0.207	−0.009	−0.130	−0.189	−0.077	−0.021	0.210	0.092	0.026	−0.039	0.065	0.026	−0.136	−0.162
HC4	0.009	0.009	0.017	0.016	0.012	0.072	−0.193	−0.169	−0.163	−0.195	−0.081	−0.175	−0.124	−0.148	−0.035	0.202	0.015	0.070	−0.067	−0.015	−0.036	−0.110	−0.114
HC5	−0.008	−0.081	0.062	0.047	0.152	0.176	−0.165	−0.168	−0.124	−0.170	0.082	−0.112	−0.189	0.068	0.117	0.243	0.169	0.221	0.177	0.126	0.293	0.055	−0.050
HC6	−0.028	0.000	0.051	0.064	−0.048	0.103	−0.177	−0.132	−0.130	−0.183	−0.042	−0.174	−0.162	−0.089	−0.002	0.158	0.003	0.144	0.006	0.032	0.055	−0.162	−0.128
HC7	0.074	0.087	0.094	0.135	0.117	0.098	−0.080	−0.044	−0.074	−0.156	0.107	−0.072	−0.241	0.009	0.020	0.269	0.164	0.401	0.274	0.166	0.243	−0.036	0.068
HC8	−0.009	−0.030	0.075	0.060	0.006	0.177	−0.167	−0.170	−0.120	−0.176	−0.002	−0.088	−0.187	−0.027	0.117	0.135	0.070	0.231	0.132	0.039	0.098	−0.137	−0.062
HC9	−0.014	−0.016	0.103	0.140	0.134	0.180	−0.101	−0.121	−0.052	−0.131	0.059	−0.146	−0.114	0.043	0.048	0.275	0.158	0.288	0.125	0.081	0.106	−0.064	0.007
HC10	0.001	−0.013	0.084	0.034	0.042	0.110	−0.180	−0.152	−0.127	−0.227	−0.005	−0.144	−0.203	−0.040	−0.013	0.140	0.117	0.199	0.131	0.058	0.113	−0.060	−0.032
HC11	−0.011	−0.006	0.093	0.116	0.000	0.193	−0.139	−0.093	−0.042	−0.155	0.019	−0.129	−0.085	0.040	0.032	0.342	0.210	0.142	0.194	0.121	−0.036	−0.070	−0.111
HC12	0.014	−0.019	0.085	0.084	−0.063	0.171	−0.199	−0.200	−0.141	−0.243	−0.154	−0.161	−0.310	−0.075	−0.087	0.175	0.118	0.059	−0.078	−0.166	−0.067	−0.264	−0.116
HC13	−0.034	−0.050	0.022	0.022	−0.016	0.131	−0.154	−0.127	−0.114	−0.170	−0.018	−0.168	−0.146	0.027	−0.049	0.153	0.095	0.169	0.144	0.080	0.085	−0.041	−0.032
**average**	**−0.003**	**−0.016**	**0.063**	**0.067**	**0.021**	**0.145**	**−0.158**	**−0.136**	**−0.117**	**−0.183**	**−0.012**	**−0.137**	**−0.187**	**−0.021**	**0.001**	**0.208**	**0.127**	**0.172**	**0.084**	**0.047**	**0.063**	**−0.092**	**−0.056**
**SD**	**0.027**	**0.042**	**0.029**	**0.043**	**0.073**	**0.040**	**0.040**	**0.041**	**0.042**	**0.036**	**0.075**	**0.040**	**0.064**	**0.070**	**0.067**	**0.068**	**0.091**	**0.109**	**0.126**	**0.088**	**0.112**	**0.079**	**0.082**
**minimum**	**−0.034**	**−0.081**	**0.017**	**0.013**	**−0.078**	**0.072**	**−0.203**	**−0.200**	**−0.186**	**−0.243**	**−0.154**	**−0.203**	**−0.310**	**−0.148**	**−0.101**	**0.121**	**0.003**	**0.026**	**−0.113**	**−0.166**	**−0.072**	**−0.264**	**−0.162**
**maximum**	**0.074**	**0.087**	**0.103**	**0.140**	**0.152**	**0.193**	**−0.080**	**−0.044**	**−0.042**	**−0.131**	**0.107**	**−0.072**	**−0.085**	**0.075**	**0.117**	**0.342**	**0.357**	**0.401**	**0.274**	**0.166**	**0.293**	**0.055**	**0.116**
