# Supplementary figures and images for: In vivo binding of a tau imaging probe, [11C]PBB3, in patients with progressive supranuclear palsy
Source: Mov Disord. 2019 Mar 20;34(5):744–54. doi: 10.1002/mds.27643 (PMC6593859; doi:10.1002/mds.27643)

PSP

eFigure 1

$BP^*_{ND}$

0.7

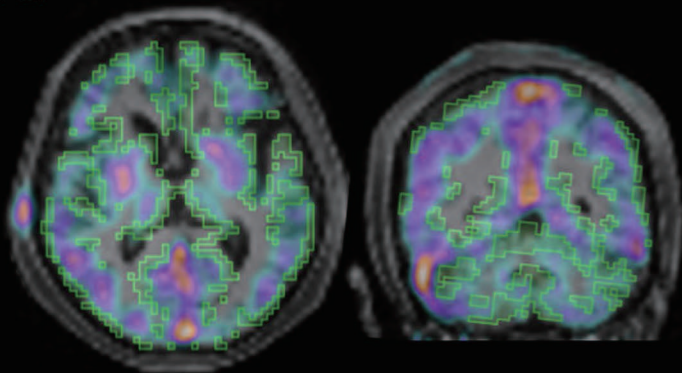

HC

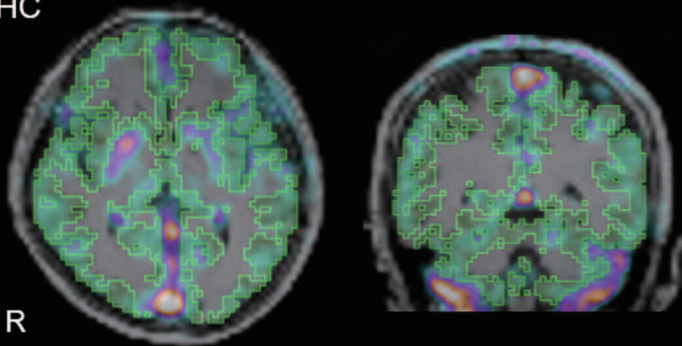

-0.2

R

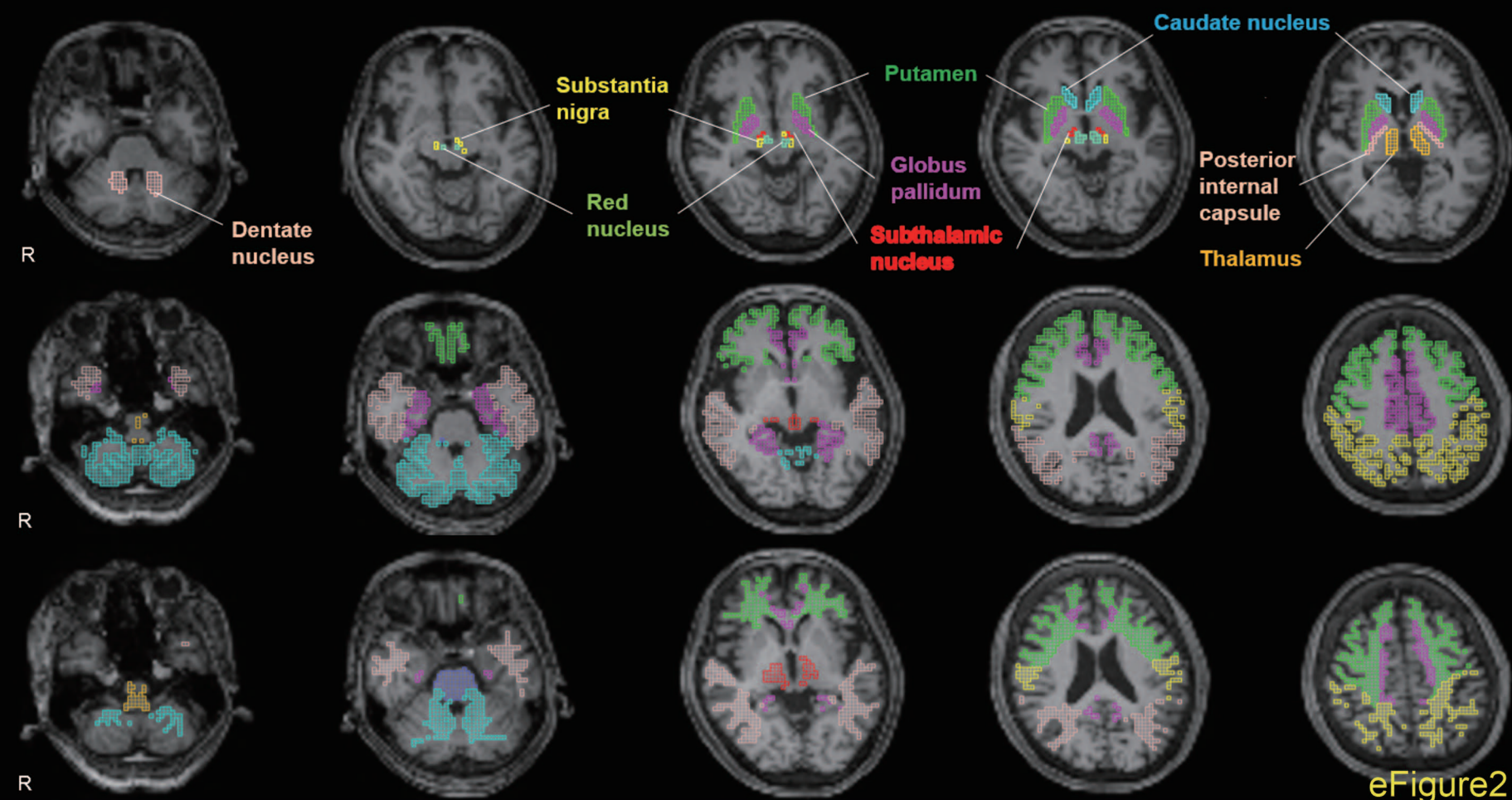

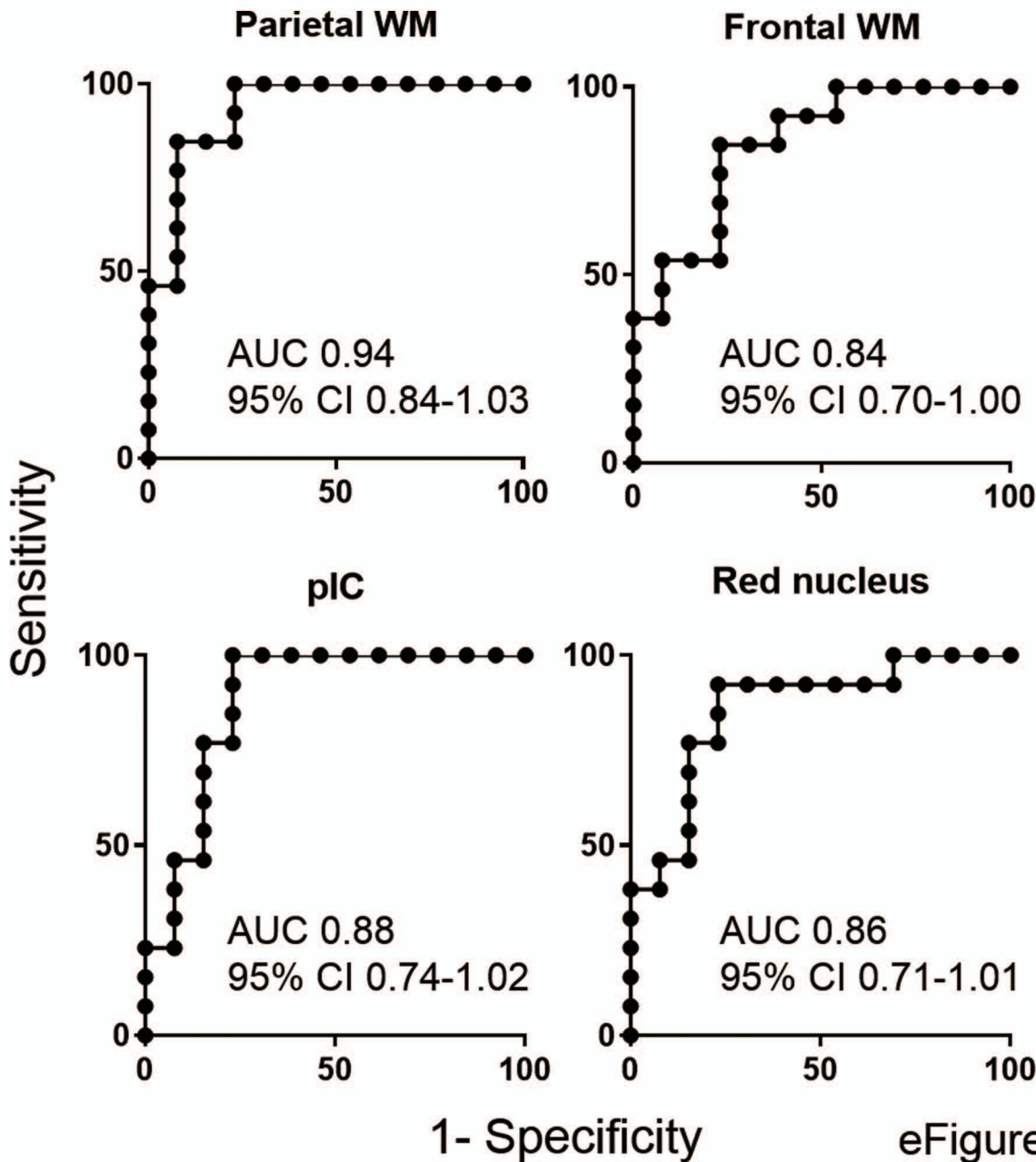

eFigure 3

Frontal WM

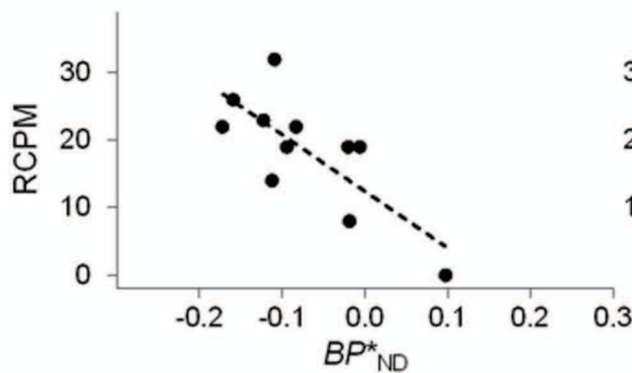

Parietal WM

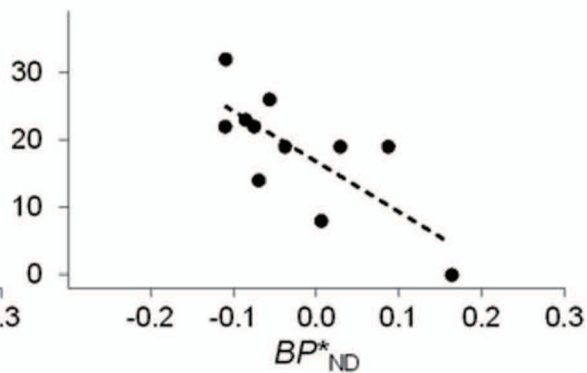

Frontal GM

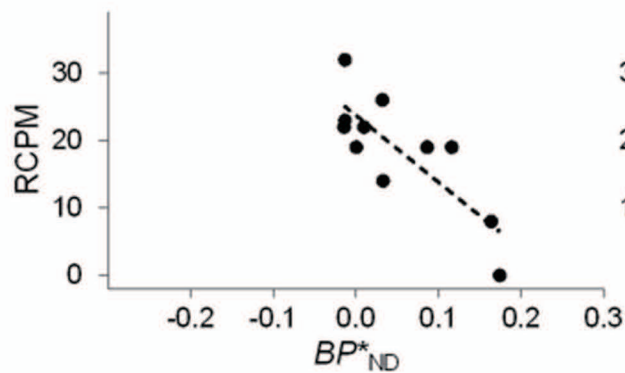

Parietal GM

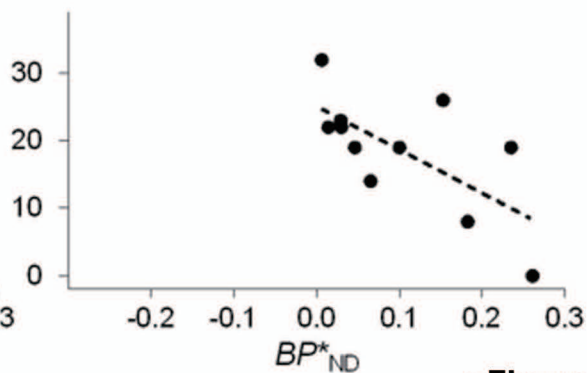

Supplement: Supplementary file 2 — Figure S1 Supporting information [file MDS-34-744-s002.pdf]
