# Supplementary material for: In vivo binding of a tau imaging probe, [11C]PBB3, in patients with progressive supranuclear palsy
Source: Mov Disord. 2019 Mar 20;34(5):744–54. doi: 10.1002/mds.27643 (PMC6593859; doi:10.1002/mds.27643)
Supplement: Supplementary file 3 — Appendix S1 Supporting information [file MDS-34-744-s003.docx]

**eMethods**

**Radiosynthesis of [^11^C]PBB3 and [^11^C]PiB**

Radiosynthesis of [^11^C]PBB3 and [^11^C]PiB was carried out as described elsewhere.^1-3^ Briefly, [^11^C]Methyl iodide reacted with tert-butyldimethylsilyl desmethyl precursor under the presence of potassium hydroxide. The final formulated product of [^11^C]PBB3 was radiochemically pure (**≥** 95%) due to the use of byanalytic high performance liquid chromatography (CAPCELL PAK C_18_ column, 4.6 mm × 250 mm; acetonitrile/50 mM ammonium formate=4/6, 2 ml/min). Specific activities of [^11^C]PBB3 and [^11^C]PiB at the time of injection were 4 – 361 and 22 – 144 GBq/μmol, respectively.

**Generation of parametric images using clustered reference voxels**

A brief description of our novel optimized reference setting method is as follows: 1) We generated binding potential (*BP**_ND_) parametric images by an original multilinear reference tissue model (MRTM_O_) using the cerebellar cortex as reference region;^2^ 2) The covering range of *BP**_ND_ for picking up reference voxels was determined as the mean full-width at half-maximum (FWHM) of a histogram generated by averaging data in separately recruited 11 young healthy subjects (mean FWHM: 0.36 ± 0.05; age: 34 ± 7 y; women/men: 6/5) who were considered to have the least tau accumulations; 3) Reference tissue was defined in gray matter (GM) of each individual as a pool of voxels that had *BP**_ND_ values above mean minus 2 standard deviations at the range of the above-mentioned mean FWHM defined in young healthy subjects; and 4) We acquired final *BP**_ND_ parametric images using MRTMo and optimized references (**eFigure 1**). There were no significant differences in *BP**_ND_ values between age-matched healthy subjects and young healthy subjects in any of the GM and white matter (WM) volumes of interest (VOIs) defined here (data not shown; for details of VOIs definition, see below).

The rationale for this approach is two-fold; unlike in Alzheimer’s disease (AD), tau aggregates are pathologically observed even in the cerebellar WM of progressive supranuclear palsy (PSP) brains.^4^ In addition, a rare subtype of PSP patients with cerebellar ataxia pathologically shows tau aggregates in cerebellar GM.^5^

In the present work, we extracted reference voxels from GM but not WM. If both GM and WM are included in the frequency histogram, WM voxels with tau pathology would be picked up as reference voxels, because non-displaceable radioligand binding in WM is lower than that of GM. An alternative option for to quantify specific binding of [^11^C]PBB3 in WM would be to extract reference voxels only from WM on the basis of a WM frequency histogram. However, the signal-to-noise ratio in WM reference tissue may not be as high as the ratio in GM reference tissue due to low non-displaceable radioligand binding in WM relative to GM. In addition, we postulate that the *BP**_ND_ in WM estimated with GM reference voxels reflects specific radioligand binding with only a constant bias across subjects, since the difference in the non-displaceable radioligand binding between tau-negative, ‘normal’ gray and WM should also be constant.

A possible drawback of the quantification with extracted reference voxels might be difficulty in picking up a sufficient number of voxels with a low likelihood of tau pathologies in a severely affected patient with extensive tau spread, although high performance of this technique was proven in AD patients with widespread tau burdens.^3^ The inclusion of tau-positive voxels in the reference tissue would also result in underestimation of *BP**_ND_ values, and therefore should not increase false positive findings. Additionally, *BP**_ND_ estimated by an original multilinear reference tissue model is equivalent to the target-to-reference ratio of radioligand retention minus 1 at an equilibrium state,^2^ and negative *BP**_ND_ values in several brain regions of HCs and PSP patients are likely to stem from the great dependency of [^11^C]PBB3 uptake on the first-pass extraction due to the rapid metabolic conversion of this compound.^1,2^

As illustrated in **eFigure 1**, a significant subset of reference voxels neighbored the brain surface or ventricles in PSP patients as compared to HCs, and radioligand retentions in these voxels could be underestimated due to partial volume effects. Meanwhile, greater partial volume effects may be caused by atrophy related to neurodegeneration in target voxels enriched with tau aggregates. In addition, reference voxels in the proximity of abundant tau pathologies might actually contain a small amount of tau deposits, leading to underestimation of radioligand binding in target voxels, as mentioned above. Despite these potential issues, our previous work on the new reference definition method demonstrated that total distribution volumes for [^11^C]PBB3 determined by a graphical analysis did not differ between reference voxels in AD and HC groups,^3^ supporting the validity of the current methodology at a practical level.

**Definition of volumes of interest on PET images**

VOIs were defined by manual and semi-automatic methods. VOIs were manually placed in the subcortical nuclei of each subject on the final *BP**_ND_ images superimposed on T1-weighted MRI images. A template atlas (Talairach Daemon atlas from the Wake Forest University PickAtlas version 3.0.5) which was extended to the vertex in MNI (Montreal Neurological Institute) space was transformed to individual MR space and used for guidance in the manual definition of VOIs.^6,7^ Combined VOIs over the two hemispheres consisted of the following areas: caudate nucleus (2.49 ± 0.11 cm^3^), putamen (6.98 ± 0.37 cm^3^), globus pallidus (2.53 ± 0.11 cm^3^), thalamus (3.53 ± 0.09 cm^3^), subthalamic nucleus (0.25 ± 0.02 cm^3^), substantia nigra (0.30 ± 0.02 cm^3^), red nucleus (0.37 ± 0.01 cm^3^), cerebellar dentate nucleus (1.76 ± 0.09 cm^3^) and posterior internal capsule (1.17 ± 0.09 cm^3^) (**eFigure 2A**). As prominent venous radioactivity in [^11^C]PBB3-PET images impedes manual definitions of other VOIs, VOIs were semi-automatically placed on frontal, parietal, temporal, limbic, midbrain, pons, medulla and cerebellar regions from Wake Forest University PickAtlas version 3.0.5,^8^ based on Talairach Daemon atlases transformed to individual space.^6,7^ In this procedure, we applied a normalization approach based on 3 tissue probability maps, which was implemented in PMOD 3.6. Subsequently, all template VOIs were inversely transformed from MNI space to individual space. To reduce any effects of brain atrophy and radioactivity spill-over from venous sinuses, all semi-automatically defined VOIs were intersected with GM and WM based on segmented T1-weighted MR images (voxels with 80% or higher probability of GM or WM were included). (**eFigure 2B, 2C**). For example, resulting ‘limbic WM’ is a WM segment in the individually transformed limbic lobe VOI. Although there seemed to be pronounced spill-in from sagittal sinus in paramedian regions (**Figure 1B**), such effects were minimal in the majority of voxels in cortical VOIs (**Figure 1A**), which was also indicated by an observation that the mean parietal GM *BP**_ND_ approximated 0 (**Figure 2B**). GM VOIs in the pons and medulla were not used for subsequent quantitative analyses, as they had very small numbers or no voxels.

**Image analyses**

All *BP**_ND_ values were obtained from individual PET data spaces. *BP**_ND_ images corrected for head motions were co-registered to individual T1-weighted MR images by using blood flow images (relative [^11^C]PBB3 tracer delivery),^9^ and then all the prepared VOIs described above were inversely transformed from T1-weighted MR space to PET space.

Analysis of [^11^C]PiB PET data was carried out as described elsewhere.^1^

**A receiver operating characteristic (ROC) curve analysis**

In order to assess the sensitivity and specificity of differentiation between age-matched HCs and PSPs by [^11^C]PBB3-PET, a ROC curve analysis of regional *BP**_ND_ values for [^11^C]PBB3 was performed. We applied "minimum distance to the upper left corner" to determine an optimal cut-off *BP**_ND_ value for separating the two diagnostic groups.

The area under the ROC curve generated with regional *BP**_ND_ values for [^11^C]PBB3 was highest in parietal gray matter and white matter, and sensitivity and specificity for the separation between PSPs and age-matched HCs were approximately 85% or higher, with an optimized cut-off *BP**_ND_ value in these areas (eTable 2). The ROC assays demonstrated the applicability of regional *BP**_ND_ values for [^11^C]PBB3 to the differentiation between PSPs and age-matched HCs with considerably high sensitivity and specificity.

**Autoradiography of PSP brain sections with [^11^C]PBB3**

Autoradiography was conducted using 6-μm-thick deparaffinized PSP brain sections. Slices were pre-incubated in 50 mM Tris-HCl buffer (pH 7.4) containing 20% ethanol at room temperature for 30 min, and then incubated in 50 mM Tris-HCl buffer (pH 7.4) containing 20% ethanol and 10 nM of [^11^C]PBB3 at room temperature for 60 min. The tissues were subsequently rinsed with ice-cold Tris-HCl buffer containing 20% ethanol twice for 2 min, and were dipped into ice-cold water for 10 sec. The sections were dried by treating with warm air and were exposed to an imaging plate (BAS-MS2025; Fuji Film, Tokyo, Japan). Imaging plates were scanned with a BAS-5000 system (Fuji Film) to obtain autoradiograms. Excess concentration (100 μM) of PBB5, an analog of PBB3,^1^ was added to the reaction to assess nonspecific radioligand binding.

**Histochemical and immunohistochemical staining of PSP brain sections**

*In-vitro* fluorescence staining of formalin-fixed, paraffin-embedded PSP brain sections adjacent to those used for autoradiography was performed with PBB3. Sections were incubated with 10 μg/ml of non-radioactive PBB3 in 50% ethanol for 30 min at 25°C, followed by washing with 50% ethanol for 5 min, as in our previous work.^1^ Fluorescence images were captured by Lecia DM4000 microscope (Leica Microsystems, Wetzlar, Germany) with a blue-violet excitation fluorescence filter unit (excitation with 391–437 nm band-pass filter and emission with 458 nm long-pass filter; manufactured by OPTO-LINE, Inc. Tokyo, Japan).

Sections were then autoclaved for antigen retrieval, and were immunohistochemically stained as described elsewhere.^10^ Briefly, the samples were reacted with a monoclonal antibody against phosphorylated tau, AT8 (1:250; Thermo Scientific, Rockford, IL). Immunoreactivity was detected by secondary Alexa Fluor 488 goat anti-mouse IgG antibody (1:500; Life Technologies, Carlsbad, CA), and was imaged by the above-mentioned microscopy with blue excitation fluorescence filter unit (Leica L5 filter set; excitation with 460–500 nm band-pass filter and emission with 512–542 nm band-pass filter).

Subsequently, Gallyas silver staining was performed as described previously.^11,12^ Bright-field images were captured by the above-mentioned microscopy.

**eFigure legends**

**eFigure 1.**

Optimized reference voxels extracted from GM of a representative PSP patient (top row) and an age-matched HC subject (bottom row). Transaxial (left) and coronal (right) *BP**_ND_ parametric images with optimized reference voxels (light green area) extracted from cerebral and cerebellar cortical GM.

**List of abbreviations**

Binding potential (*BP**_ND_: a binding parameter that directly reflects tau density), healthy control (HC), gray matter (GM), progressive supranuclear palsy (PSP), white matter (WM)

**eFigure 2.**

**A: Manual definition of subcortical VOIs**

VOIs were placed on the caudate nucleus (light blue), putamen (green), globus pallidus (pink), thalamus (orange), subthalamic nucleus (red), substantia nigra (yellow), red nucleus (light green), cerebellar dentate nucleus (beige) and posterior internal capsule (beige).

**B and C: Semi-automatic definition of GM and WM VOIs**

GM and WM segmented VOIs were placed on the frontal (green), parietal (yellow), limbic (pink), temporal (beige) cortices, midbrain (red), pons (purple), cerebellum (light blue), and medulla (orange). Note that GM VOIs in the pons and medulla were not employed for quantitative analyses, as they had very small numbers or no voxels.

**List of abbreviations**

Gray matter (GM), volumes of interest (VOIs), white matter (WM)

**eFigure 3.**

**Representative receiver operating characteristic (ROC) curves for the diagnosis of PSP**

ROC analyses were conducted using [^11^C]PBB3 *BP**_ND_ in parietal and frontal white matter (WM), posterior internal capsule (pIC), and red nucleus.

**eFigure 4.**

**Correlations between PET-detectable tau deposition and clinical symptoms**

Negative correlations between non-verbal cognitive function (RCPM scores) and *BP**_ND_ in PSP patients.

Scatter plots of *BP*_ND_* in frontal GM (*r_s_* = -0.8, *P* = .004) and WM (*r_s_* = -0.7, *P* = .02) and parietal GM (*r_s_* = -0.7, *P* = .02) and WM (*r_s_* = -0.7, *P* = .01) VOIs against RCPM scores in PSP patients. Dashed lines represent regressions.

**List of abbreviations**

Binding potential (*BP**_ND_: a binding parameter that directly reflects tau density), gray matter (GM), progressive supranuclear palsy (PSP), Raven’s colored progressive matrices (RCPM), volumes of interest (VOIs), unified Parkinson’s disease rating scale part III (UPDRS), white matter (WM)

**eTable 1: [^11^C]PBB3 *BP**_ND_: mean (SD) and effect sizes corresponding to Figure 2**

| VOIs | HC *BP**_ND_ | | | | PSP *BP**_ND_ | | | | Effect size (η^2^) | Z score |
| --- | --- | --- | --- | --- | --- | --- | --- | --- | --- | --- |
|  | Mean | SD | MIN | MAX | Mean | SD | MIN | MAX |  |  |
| Caudate | 0.00 | 0.07 | -0.10 | 0.12 | 0.02 | 0.10 | -0.11 | 0.22 | 0.01 | 0.22 |
| Putamen | 0.21 | 0.07 | 0.12 | 0.34 | 0.28 | 0.09 | 0.13 | 0.43 | 0.18 | 1.08 |
| Globus pallidus | 0.13 | 0.09 | 0.00 | 0.36 | 0.25 | 0.09 | 0.08 | 0.39 | 0.31 | 1.31 |
| Thalamus | 0.17 | 0.11 | 0.03 | 0.40 | 0.30 | 0.12 | 0.15 | 0.53 | 0.24 | 1.13 |
| Subthalamic nucleus | 0.08 | 0.13 | -0.11 | 0.27 | 0.27 | 0.15 | 0.09 | 0.60 | 0.33 | 1.51 |
| Substantia nigra | 0.05 | 0.09 | -0.17 | 0.17 | 0.14 | 0.13 | -0.09 | 0.40 | 0.17 | 1.10 |
| Red nucleus | 0.06 | 0.11 | -0.07 | 0.29 | 0.24 | 0.14 | 0.00 | 0.50 | 0.34 | 1.57 |
| Cerebellar dentate nucleus | -0.09 | 0.08 | -0.26 | 0.06 | 0.00 | 0.07 | -0.11 | 0.10 | 0.30 | 1.16 |
| Posterior internal capsule | -0.06 | 0.08 | -0.16 | 0.12 | 0.08 | 0.09 | -0.02 | 0.25 | 0.42 | 1.72 |
| Frontal GM | 0.00 | 0.03 | -0.03 | 0.07 | 0.04 | 0.07 | -0.04 | 0.17 | 0.18 | 1.75 |
| Parietal GM | -0.02 | 0.04 | -0.08 | 0.09 | 0.10 | 0.09 | 0.01 | 0.26 | 0.43 | 2.75 |
| Temporal GM | 0.06 | 0.03 | 0.02 | 0.10 | 0.10 | 0.06 | 0.01 | 0.22 | 0.17 | 1.43 |
| Limbic GM | 0.07 | 0.04 | 0.01 | 0.14 | 0.09 | 0.06 | 0.00 | 0.22 | 0.06 | 0.62 |
| Cerebellar GM | 0.14 | 0.04 | 0.07 | 0.19 | 0.16 | 0.07 | 0.07 | 0.35 | 0.02 | 0.34 |
| Midbrain GM | 0.02 | 0.07 | -0.08 | 0.15 | 0.07 | 0.14 | -0.11 | 0.31 | 0.05 | 0.70 |
| Frontal WM | -0.16 | 0.04 | -0.20 | -0.08 | -0.07 | 0.07 | -0.17 | 0.10 | 0.36 | 2.10 |
| Parietal WM | -0.14 | 0.04 | -0.20 | -0.04 | -0.02 | 0.08 | -0.11 | 0.16 | 0.45 | 2.72 |
| Temporal WM | -0.12 | 0.04 | -0.19 | -0.04 | -0.06 | 0.07 | -0.15 | 0.07 | 0.20 | 1.24 |
| Limbic WM | -0.18 | 0.04 | -0.24 | -0.13 | -0.12 | 0.05 | -0.19 | 0.02 | 0.30 | 1.65 |
| Cerebellar WM | -0.02 | 0.07 | -0.15 | 0.08 | 0.00 | 0.05 | -0.07 | 0.10 | 0.04 | 0.35 |
| Midbrain WM | -0.01 | 0.07 | -0.15 | 0.11 | 0.07 | 0.10 | -0.09 | 0.24 | 0.20 | 1.12 |
| Pons WM | -0.14 | 0.04 | -0.20 | -0.07 | -0.10 | 0.09 | -0.27 | 0.05 | 0.06 | 0.87 |
| Medullar WM | -0.19 | 0.06 | -0.31 | -0.09 | -0.18 | 0.07 | -0.38 | -0.09 | <.001 | 0.04 |

List of abbreviations

Binding potential (*BP**_ND_: binding parameter that directly reflects tau density), gray matter (GM), healthy control (HC), maximum (MAX), minimum (MIN), progressive supranuclear palsy (PSP), standard deviation (SD), volumes of interest (VOIs), white matter (WM)

**eTable 2: Regions of significantly high sensitivity and specificity based on receiver operating characteristic analysis**

| VOIs | AUC | Asymptotic significance | Sensitivity | Specificity | Cut off | FDR  *q* value |
| --- | --- | --- | --- | --- | --- | --- |
| Parietal GM | 0.95 | <.001 | 92.3 | 92.3 | 0.012 | .001 |
| Parietal WM | 0.93 | <.001 | 84.6 | 92.3 | -0.089 | .001 |
| Posterior internal capsule | 0.88 | .001 | 100.0 | 76.9 | -0.028 | .004 |
| Red nucleus | 0.86 | .002 | 92.3 | 76.9 | 0.107 | .007 |
| Frontal WM | 0.85 | .003 | 84.6 | 76.9 | -0.131 | .008 |
| Limbic WM | 0.85 | .003 | 69.2 | 84.6 | -0.149 | .007 |
| Cerebellar dentate nucleus | 0.83 | .004 | 76.9 | 84.6 | -0.035 | .007 |
| Globus pallidus | 0.80 | .01 | 69.2 | 84.6 | 0.182 | .02 |
| Subthalamic nucleus | 0.80 | .01 | 61.5 | 92.3 | 0.214 | .01 |
| Thalamus | 0.79 | .01 | 76.9 | 61.5 | 0.199 | .02 |
| Midbrain WM | 0.76 | .03 | 76.9 | 76.9 | 0.026 | .03 |
| Temporal WM | 0.74 | .04 | 76.9 | 69.2 | -0.113 | .04 |
| Putamen | 0.73 | .04 | 53.8 | 92.3 | 0.282 | .04 |

**List of abbreviations**

Area under the curve (AUC), false discovery rate (FDR), gray matter (GM), volumes of interest (VOIs), white matter (WM)

**eTable 3 Correlations of regional *BP**_ND_ of [^11^C]PBB3 with scores of RCPM and UPDRS in PSP patients.**

| Test/scale | VOIs | Correlation coefficient | *P* value | FDR *q* value |
| --- | --- | --- | --- | --- |
| RCPM | Frontal GM | -0.8 | .004 | .02 |
|  | Parietal WM | -0.7 | .01 | .04 |
|  | Parietal GM | -0.7 | .02 | .03 |
|  | Frontal WM | -0.7 | .02 | .04 |
| UPDRS | Parietal WM | 0.6 | .03 | .04 |
|  | Frontal WM | 0.6 | .05 | .05 |

**List of abbreviations**

Binding potential (*BP**_ND_: binding parameter that directly reflects tau density), false discovery rate (FDR), gray matter (GM), Raven's colored progressive matrices (RCPM), volumes of interest (VOIs), white matter (WM), unified Parkinson's disease rating scale motor score (UPDRS)

**eTable 4: Significant correlations between *BP**_ND_ and subcategories of PSPRS scores**

| Subcategories of PSPRS scores (n = 9) | VOIs | | Correlation coefficient | | *P* value | FDR　 *q* value |
| --- | --- | --- | --- | --- | --- | --- |
| Using knife and fork, buttoning clothes, washing hands and face | Frontal GM | | 0.7 | | 0.02 | 0.08 |
|  | Parietal GM | | 0.8 | | 0.02 | 0.06 |
|  | Temporal GM | | 0.7 | | 0.04 | 0.06 |
|  | Frontal WM | | 0.7 | | 0.04 | 0.06 |
|  | Parietal WM | | 0.8 | | 0.01 | 0.08 |
|  | Temporal WM | | 0.9 | | 0.004 | 0.06 |
| Falls | Midbrain WM | | 0.7 | | 0.05 | 0.06 |
| Dysarthria | Parietal GM | | 0.7 | | 0.05 | 0.06 |
| Voluntary left and right command movement | Frontal GM | | 0.7 | | 0.03 | 0.07 |
|  | Frontal WM | | 0.7 | | 0.04 | 0.05 |
|  | Limbic WM | | 0.8 | | 0.01 | 0.05 |
| Finger tapping | Frontal WM | | 0.7 | | 0.03 | 0.06 |
|  | Temporal WM | | 0.7 | | 0.03 | 0.05 |
| Apraxia of hand movement | Frontal GM | | 0.7 | | 0.05 | 0.05 |
|  | Frontal WM | | 0.7 | | 0.05 | 0.05 |
|  | Limbic WM | | 0.8 | | 0.02 | 0.05 |
|  | |  | | Spearman's rho test | |  |

**List of abbreviations**

Binding potential (*BP**_ND_: binding parameter that directly reflects tau density), false discovery rate (FDR), gray matter (GM), progressive supranuclear palsy rating scale (PSPRS), volumes of interest (VOIs), white matter (WM)

**References**

1. Maruyama M, Shimada H, Suhara T, et al. Imaging of tau pathology in a tauopathy mouse model and in Alzheimer patients compared to normal controls. *Neuron.* 2013;79(6):1094-1108.

2. Kimura Y, Ichise M, Ito H, et al. PET Quantification of Tau Pathology in Human Brain with ^11^C-PBB3. *J Nucl Med.* 2015;56(9):1359-1365.

3. Kimura Y, Endo H, Ichise M, et al. A new method to quantify tau pathologies with ^11^C-PBB3 PET using reference tissue voxels extracted from brain cortical gray matter. *EJNMMI Res.* 2016;6(1):24.

4. Williams DR, Holton JL, Strand C, et al. Pathological tau burden and distribution distinguishes progressive supranuclear palsy-parkinsonism from Richardson's syndrome. *Brain.* 2007;130(6):1566-1576.

5. Kanazawa M, Shimohata T, Toyoshima Y, et al. Cerebellar involvement in progressive supranuclear palsy: A clinicopathological study. *Mov Disord.* 2009;24(9):1312-1318.

6. Lancaster JL. The Talairach Daemon, a database server for Talairach Atlas Labels. *NeuroImage.* 1997; 5:S633.

7. Lancaster JL, Woldorff MG, Parsons LM, et al. Automated Talairach atlas labels for functional brain mapping. *Hum Brain Mapp*. 2000;10(3):120-131.

8. Maldjian JA, Laurienti PJ, Kraft RA, Burdette JH. An automated method for neuroanatomic and cytoarchitectonic atlas-based inter- rogation of fMRI data sets. *Neuroimage.* 2003;19(3):1233-1239.

9. Ichise M, Liow JS, Lu JQ, et al. Linearized reference tissue parametric imaging methods: application to [^11^C]DASB positron emission tomography studies of the serotonin transporter in human brain. *J Cereb Blood Flow Metab.* 2003;23(9):1096-1112.

10. Maeda J, Zhang MR, Okauchi T, Ji B, Ono M, Hattori S, et al. In vivo positron emission tomographic imaging of glial responses to amyloid-beta and tau pathologies in mouse models of Alzheimer's disease and related disorders. *J Neurosci.* 2011;31(12):4720-4730.

11. Uchihara T, Kondo H, Kosaka K, Tsukagoshi H. Selective loss of nigral neurons in Alzheimer’s disease: a morphometric study. *Acta Neuropathol.* 1992; 83(3): 271-276.

12. Uchihara T. Silver diagnosis in neuropathology: principles, practice and revised interpretation. *Acta Neuropathol.* 2007;113(5):483-499.
